# Sugar beet root susceptibility to storage rots and downregulation of plant defense genes increases with time in storage

**DOI:** 10.1038/s41598-024-78323-4

**Published:** 2024-11-08

**Authors:** Shyam L. Kandel, John D. Eide, Andrea Firrincieli, Fernando L. Finger, Abbas M. Lafta, Karen K. Fugate

**Affiliations:** 1 Edward T. Schafer Agricultural Research Center, Sugarbeet Research Unit, USDA-ARS, Fargo, ND 58102 USA; 2 Department for Innovation in Biological, Agro-Food and Forest Systems, University of Tuscia, Viterbo, Italy; 3 Departamento de Agronomia, Universidade Federal de Vicosa, 36570-900 Vicosa, MG Brazil; 4 Department of Plant Pathology, North Dakota State University, P.O. Box 6050, Fargo, ND 58108 USA

**Keywords:** *Botrytis*, Pathogen receptor, Phytohormone signaling, *Penicillium*, Storage, Sugar beet, Molecular biology, Plant sciences

## Abstract

Storage rots are a significant cause of postharvest losses for the sugar beet crop, however, intrinsic physiological and genetic factors that determine the susceptibility of roots to pathogen infection and disease development are unknown. Research, therefore, was carried out to evaluate the disease development in sugar beet roots caused by two common storage pathogens as a function of storage duration and storage temperature, and to identify changes in the expression of defense genes that may be influencing the root susceptibility to disease. To evaluate root susceptibility to disease, freshly harvested roots were inoculated with *Botrytis cinerea* or *Penicillium vulpinum* on the day of harvest or after 12, 40, or 120 d storage at 5 or 12 °C and the weight of rotted tissue present in the roots after incubation for 35 d after inoculation were determined. Disease susceptibility and progression to *B. cinerea* and *P. vulpinum* increased with storage duration with elevations in susceptibility occurring more rapidly to *B. cinerea* than *P. vulpinum*. Also, *B. cinerea* was more aggressive than *P. vulpinum* and caused greater rotting and tissue damage in postharvest sugar beet roots. Storage temperature had minimal effect on root susceptibility to these rot-causing pathogens. Changes in defense gene expression were determined by sequencing mRNA isolated from uninoculated roots that were similarly stored for 12, 40 or 120 d at 5 or 12 °C. As susceptibility to rot increased during storage, concurrent changes in defense-related gene expression were identified, including the differential expression of 425 pathogen receptor and 275 phytohormone signal transduction pathway-related genes. Furthermore, plant resistance and hormonal signaling genes that were significantly altered in expression coincident with the change in root susceptibility to storage rots were identified. Further investigation into the function of these genes may ultimately elucidate methods by which storage rot resistance in sugar beet roots may be improved in the future.

**Supplementary Information:**

The online version contains supplementary material available at 10.1038/s41598-024-78323-4.

## Introduction

Sugar beet (*Beta vulgaris* L.) taproots contain up to 21% sucrose by fresh weight and are used to manufacture sugar on an industrial scale^1,2^. The Red River Valley (RRV) of Minnesota and North Dakota leads the United States in sugar beet production, contributing nearly 58% of total domestic production. Due to high tonnage of the crop that exceeds immediate sugar factory processing capabilities, postharvest sugar beet roots are often stored for many weeks before processing. In the RRV of Minnesota and North Dakota, sugar beet roots are stored in large outdoor piles or ventilated sheds for up to 280 d prior to processing^3^. During storage, it is estimated that respiration causes 50–80% sucrose loss^4–6^. Furthermore, elevated temperature and free moisture inside the storage piles promote microbial activities leading to the rapid deterioration and softening of root tissues^7,8^. Also, sugar beet roots are prone to mechanical damage during leaf removal, harvesting, and loading and reloading of roots during transportation and storage. Damaged areas of the root are susceptible to disease since they provide microbes access to internal root tissue.

Several fungi and bacteria can cause storage rots or postharvest diseases and sugar loss in sugar beet in the U.S. and elsewhere^7,9–14^. *Botrytis cinerea* and *Penicillium* spp. are reported as major fungal pathogens associated with storage rots in sugar beet^7^. *B. cinerea* and *Penicillium* species are necrotrophic fungi which can infect and cause postharvest losses in many agricultural crops including sugar beet^11,14–16^. Both pathogens require exposed tissues (e.g., wounds, cracks, or bruises) for infection and colonization of root tissue. Cell wall degrading enzymes and toxins secreted by *Botrytis* and *Penicillium* pathogens facilitate the initial stage of infection and subsequent colonization^17–19^. Following colonization, infected tissue collapses and rotted, moldy disease symptoms occur which can be easily noticed by the naked eye. In general, abundant fungal biomass and spore masses are produced in the decaying area of infected sites. Plant defense mechanisms utilized by sugar beet roots to limit storage rots and the factors affecting these mechanisms still need to be determined.

Presently, host resistance in sugar beet cultivars to limit storage diseases and sucrose losses has been largely unexplored due to a lack of knowledge regarding the genetic and molecular basis of defense responses during the storage. Host resistance has been utilized to minimize the damage caused by Cercospora leaf spot or Rhizomania disease in sugar beet^20,21^, but no genes that regulate or contribute to immune responses or pathogen resistance in postharvest sugar beet roots have been identified. In plants, pathogen receptor proteins and phytohormones play significant roles in the provision of protection from abiotic and biotic stresses^22–25^ and are likely to influence disease development in stored sugar beet roots. RNA sequencing (RNA-seq) technology has enabled study of the genome-wide expression of stress and disease responsive genes in a variety of crop plants^26–30^. In this study, we determined the effect of storage time and temperature on the severity of postharvest rots caused by the common storage pathogens; *B. cinerea* and *P. vulpinum*. We also identified pathogen receptor and phytohormone signal transduction genes that were altered in expression with respect to storage with a potential role in host resistance. To the best of our knowledge, this is the first report of genome-wide transcriptional changes in defense-related genes that are altered in expression during storage of sugar beet roots.

## Materials and methods

### Plant materials, storing of sugar beet roots, pathogen inoculation, and disease assessment

Sugar beets (variety VDH66156, SESVanderHave, Tienen, Belgium) were grown in a greenhouse in 15 L pots with 16 h light/8 h dark periods as previously described^31^. Roots were harvested from 16 to 17 weeks old plants. All leaf material was removed, and roots were washed gently to remove adhering potting mix. On the day of harvest (0 d), eight randomly selected roots were inoculated with *Botrytis cinerea*, and an additional eight randomly selected roots were inoculated with *Penicillium vulpinum* (formerly *P. claviforme*^32^) to evaluate susceptibility to storage rot from these two pathogens using assays described below. Pathogen cultures were obtained from diseased roots collected from commercial sugar beet piles by W. Bugbee (USDA-ARS, Fargo, ND, retired). The remaining roots were randomly divided into two groups. One group was stored at 5 °C and the other group was stored at 12 °C, with roots at both temperatures stored at 95% relative humidity. After 12 d in storage, eight random roots from each storage temperature were inoculated with *B. cinerea* and an additional eight random roots per storage temperature were inoculated with *P. vulpinum* for rot susceptibility assays. Similar operations of roots and inoculations were carried out after 40 and 120 d using roots stored at both 5 and 12 °C.

Roots were inoculated and assessed for their susceptibility to storage rot from *B. cinerea* or *P. vulpinum* using the protocol of Fugate et al.^33^. Two holes, 12 × 10 mm (diameter x depth), were drilled on opposing sides of each taproot where root girth was greatest. A mycelia-covered agar plug (10 mm diameter), cultured as previously described^33^, was inserted into each hole, with mycelia facing the exposed root tissue at the base of the hole. Following inoculation, roots were incubated in a 20 °C, 95% relative humidity growth chamber (Conviron, model PGR15). After five weeks incubation, susceptibility to rot was evaluated by excising and weighing the rotted tissue from each inoculation site. Weight of the excised tissue from the two inoculation sites per root were averaged to generate a single value for each root. The experiment was conducted with eight replications for each storage temperature x storage time combination, with individual roots as the experimental unit. Roots inoculated with *B. cinerea* were evaluated independently from roots inoculated with *P. vulpinum*, and the experiment was repeated three times. The analysis of variance (ANOVA) with Fisher’s LSD was used to identify temperature and storage duration treatments that differed significantly.

## Storage experiments and sample collections

In a separate experiment, taproots were harvested from 42 plants, and used to evaluate storage-related transcriptional changes in sugar beet roots. Tissue samples were collected individually from six roots on the day of harvest. Roots were longitudinally sectioned into four quarters and the tissue from one quarter section of each root, which was representative of the entire root (root crown to root tail) including epidermal tissue and the central vascular cylinder was collected. Tissue samples were rapidly frozen in liquid nitrogen, lyophilized, ground to a fine powder, and stored at -80 °C prior to use. The remaining roots were randomly divided into two groups which were stored in the two chambers of a Conviron (Winnipeg, MB, Canada) E7/2 two-tier growth chamber, with one chamber operating at 5 °C, the other operating at 12 °C, and both chambers set to 95% relative humidity. Six roots were randomly removed from each chamber after 12, 40, and 120 d in storage and tissue samples were collected from these roots as described above. The experiment was conducted with six replications for each storage temperature x storage time combination, with individual roots as the experimental unit. Sugarbeet plants used in our experiments were grown in the greenhouse as per USDA-ARS guidelines and comply with relevant institutional, national, and international guidelines and legislation.

## RNA isolation, sequencing, and data analysis

Total RNA was extracted from lyophilized sugar beet root tissue using a RNeasy Plant Mini Kit (QIAGEN, Valencia, CA) with an on-column DNase digestion. An entire root section was pulverized to a very fine powder, mixed well, and 50 mg of pulverized powder was used for the RNA extraction. RNA concentration was quantified using a ThermoFisher Scientific NanoDrop ND-1000 (Waltham, MA) and RNA integrity was confirmed using an Agilent Technologies 2100 Bioanalyzer (Pal Alto, CA). RNA was fragmented, converted to cDNA using random primers, amplified by PCR, and sequenced by BGI Americas (Cambridge, MA) using a BGISEQ-500 sequencing system, DNA nanoball technology, and 100 bp paired-end sequencing. An average of 29.3 M raw reads was generated per sample. Sequencing reads were processed using SOAPnuke ver. 1.5.2^34^ with > 28 million high-quality (HQ) reads per sample. HQ reads were mapped to the sugar beet genome^35^ using Bowtie2 ver. 2.2.5^36^ and gene expression levels were calculated with RSEM version 1.2.12^37^. Differentially expressed genes (adjusted-p-value < 0.01 and absolute log_2_ fold change > 1.0) were detected using DEseq2^38^.

## Identification of differentially expressed plant resistance and phytohormone related genes

The Pathogen Receptor Genes database (PRGdb: http://prgdb.org/prgdb4/)^39^ was used to identify pathogen receptor genes in sugar beet that were differentially expressed during storage (5 or 12 °C for 12, 40, or 120 d versus 0 d). Specifically, *B. vulgaris* aliases (BVRB_*) identified as pathogen receptor genes were manually downloaded from PRGdb and used to recover the corresponding Entrez gene ID from the National Center for Biotechnology Information (NCBI) database^39,40^. PCA was performed to display the direction of variability in differential expression of pathogen receptor genes and visualized using the factoextra R package^41^.

The DEGs involved in the phytohormone signal transduction pathways were recovered by using the functional annotation based on KEGG database and identified KEGG orthologs involved in ABA, auxin, BR, CTK, ET, GA, JA, and SA signaling pathways^42,43^.

Upset plots and heatmaps for significantly differentially expressed (up-and down-regulated) genes were generated using the ComplexHeatmap R package^44^.

## Results

### Storage duration and temperature effects on disease susceptibility

**Fig. 1:**
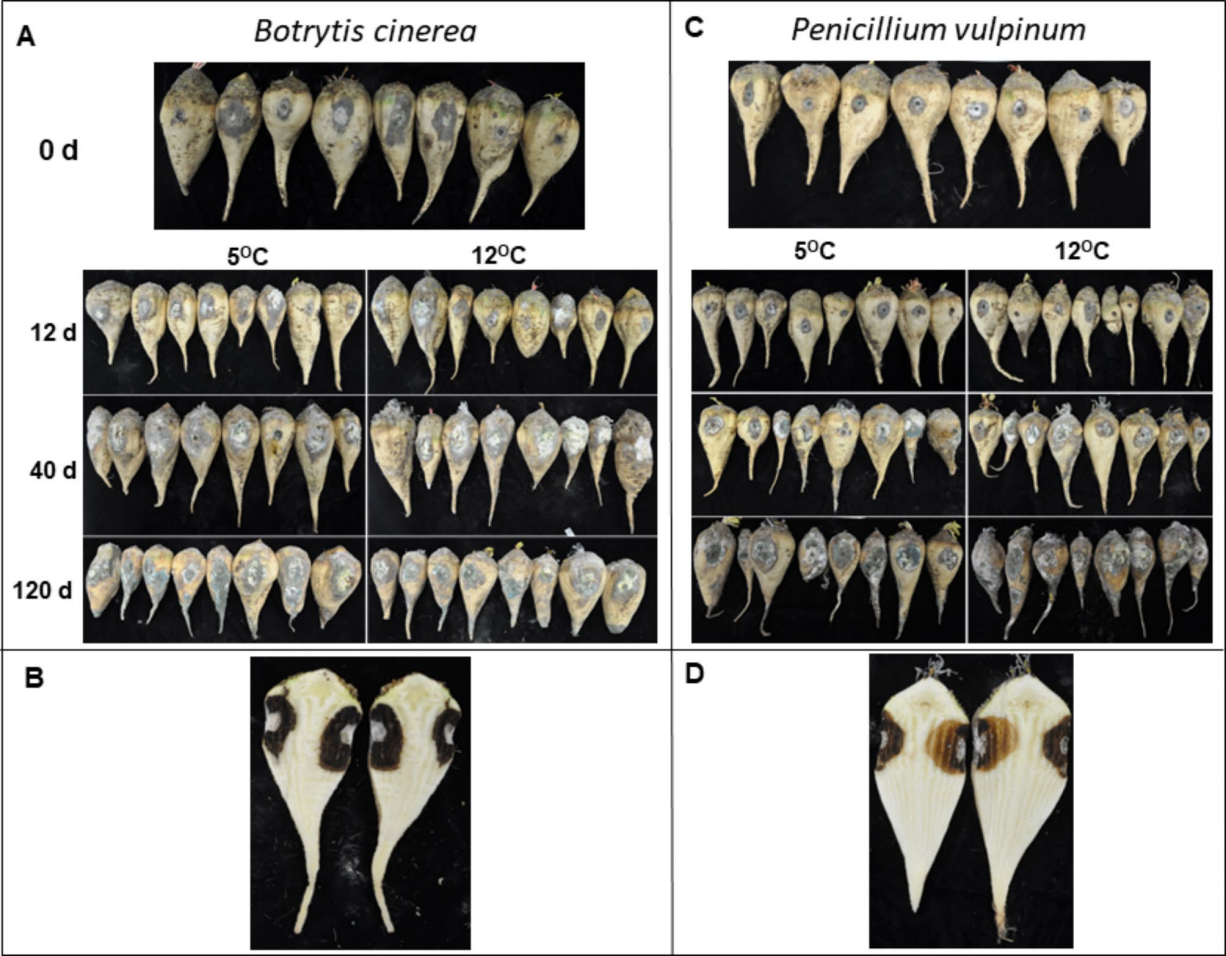
Rot symptoms on sugar beet roots caused by (**A**) *Botrytis cinerea* and (**C**) *Penicillium vulpinum.* Longitudinal sections of sugar beet roots displaying internal rot symptoms of *B. cinerea* (**B**) and *P. vulpinum* (**D**). Sugar beet roots were stored for 0, 12, 40, and 120 d at 5 or 12 °C, after which they were inoculated and incubated for 35 d at 20 °C and 95% relative humidity.

**Fig. 2:**
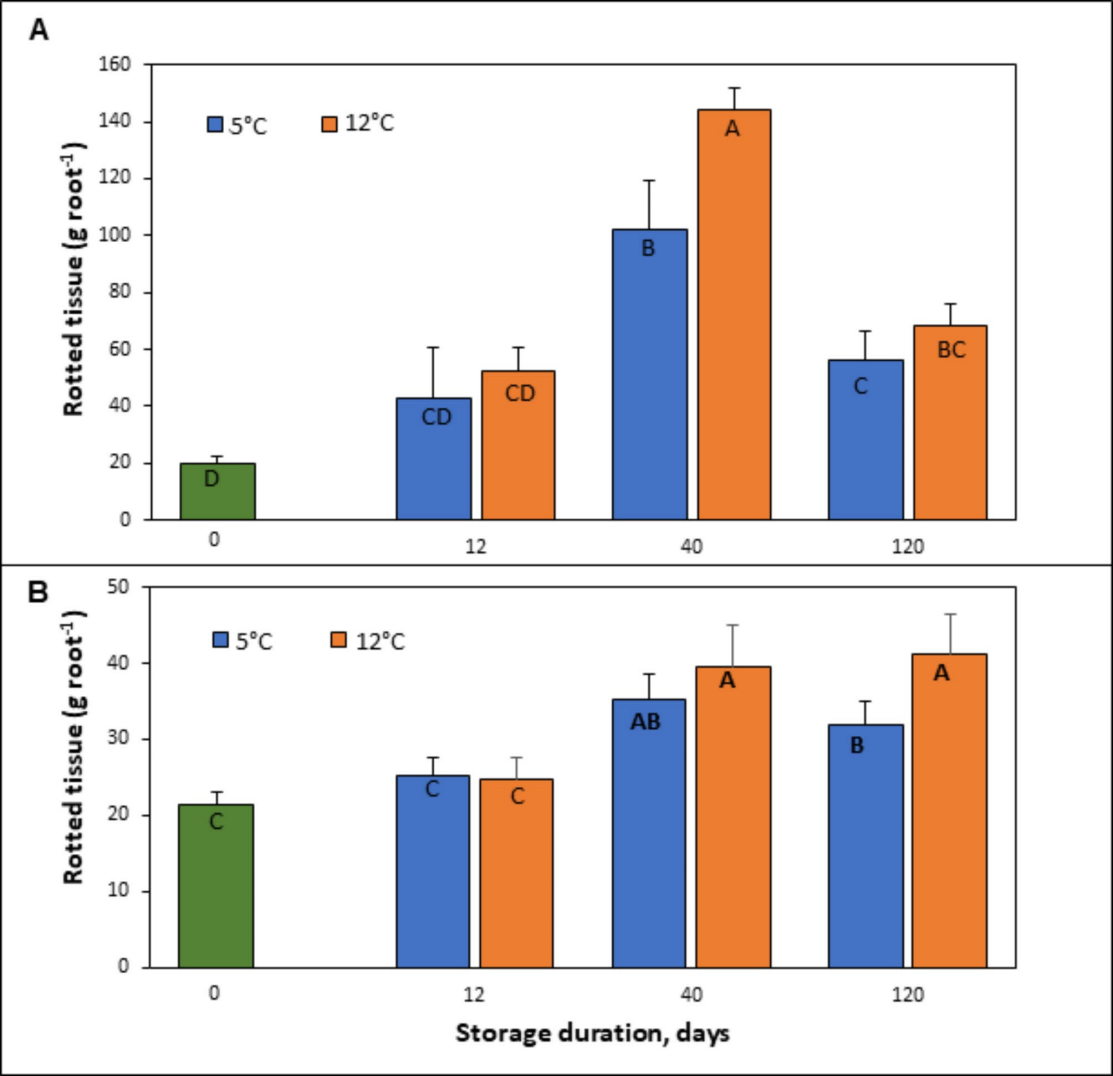
Fresh weight of rotted tissue in sugar beet roots inoculated with (**A**) *Botrytis cinerea* and (**B**) *Penicillium vulpinum.* The weight of rotted tissue was measured at 35 d after pathogen inoculation. Means that do not share a letter are significantly different (*P* < 0.05) according to Fisher’s Least Significant Difference (LSD) Method. Vertical error bars represent standard error of means.

*B. cinerea* and *P. vulpinum* infected and caused rotting symptoms in sugar beet roots that were inoculated with these pathogens at 0 d or after storage for 12, 40, and 120 d at 5 or 12 °C (Figs. 1 and 2). Both pathogens caused significantly greater rotting symptoms in roots that were stored for 40 or 120 d compared to 0 or 12 d, indicating that root defenses against the fungal pathogens diminished with storage duration. After 40 d storage at 12^°^C, *B. cinerea* inoculated roots had significantly more rotted tissue compared to roots inoculated on 0, 12 or 120 d at 5 and 12^°^C post storage (Fig. 2). In *P. vulpinum* inoculated roots, significantly more rotted tissue was observed at 40 and 120 d post storage than at 0 and 12 d, and the maximum weight of rotted tissues was observed in roots that were inoculated after storage for 120 d at 12 °C. Rotted lesions were generally larger in roots inoculated with *B. cinerea* than roots inoculated with *P. vulpinum* (Fig. 1). Furthermore, both pathogens caused the characteristic blackish-brownish discoloration in the internal root tissues at inoculation sites (Fig. 1). In general, storage temperature had minimal effect on the susceptibility of roots to storage rot caused by either pathogen. No significant difference in the weight of damaged root tissue was found between roots stored at 5 and 12^°^C for either pathogen except for *B. cinerea*-infected roots inoculated after 40 d or *P. vulpinum*-inoculated roots after 120 d (Fig. 2). Results of disease assays indicate that susceptibility to *B. cinerea* and *P. vulpinum* increases with storage duration, with rot symptoms of greater severity with *B. cinerea* than *P. vulpinum*, but minimal at higher temperature.

## Storage duration and temperature effects on expression of plant pathogen receptor genes

**Fig. 3:**
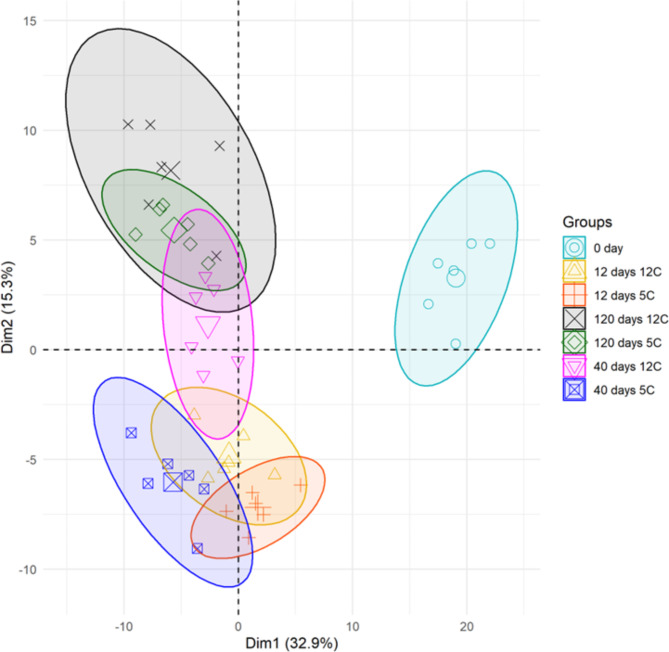
PCA plot of differentially expressed pathogen receptor genes in sugar beet roots during storage. The percentage of variation on the first and second axes was calculated based on log_2_ transformed FPKM expression data across treatments at 0, 12, 40, and 120 d of storage for roots stored at 5 or 12 °C. The 95% confidence ellipses were inserted covering six replicates of each treatment.

**Fig. 4:**
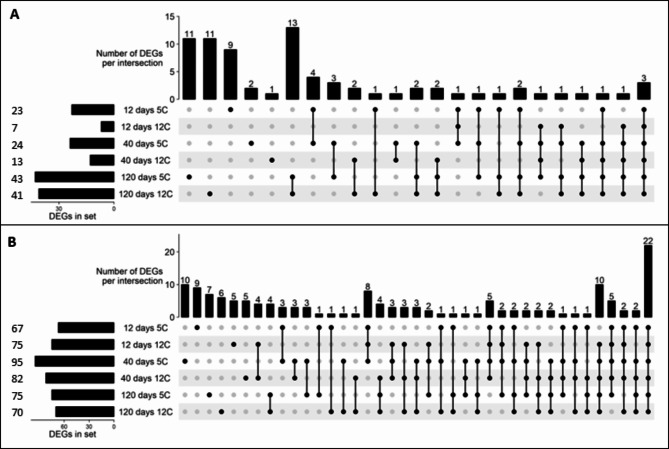
UpSet plots of significantly up (**A**) and down (**B**) regulated pathogen receptor genes (absolute log_2_fold change > 2.0 and P-adj < 0.01) in stored sugar beet roots. Total counts of up-and down-regulated genes in each treatment were displayed as horizontal bars, located on the left side of each panel. Vertical black lines with filled dots indicate sets of intersections between two or more treatments. Vertical bars in the upper portion of each panel represent total counts of unique or shared genes across treatments. The list of up-and-down regulated genes was included in Supplementary File 2.

Differentially expressed pathogen receptor genes were identified by RNA sequencing of sugar beet roots that were freshly harvested (0 day) and stored for 12, 40 or 120 d at 5 and 12°C. A total of 425 pathogen receptor genes were found differentially expressed in stored sugar beet roots (Supplementary File 1). A principal component analysis (PCA) of differentially expressed genes (DEGs) indicates a clear difference in the expression of pathogen receptors in stored versus freshly harvested roots (Fig. 3). The storage time-dependent clustering highlights a shift in the expression of pathogen receptor genes in stored roots at 12, 40, and 120 d (Fig. 3). At 12 and 40 d, distinct clustering of these genes between two storage temperatures, 5 and 12°C was observed, but no such difference was found at 120 d (Fig. 3). A total of 75 and 149 pathogen receptor genes were significantly up-and down-regulated (absolute log_2_fold change > 2.0 and P-adj < 0.01), respectively, in stored sugar beet roots (Fig. 4A-B). Nearly 75% of significantly expressed receptor genes were down-regulated as a general response to storage (Fig. 4), with 22 genes down-regulated across all storage treatments (Fig. 4B). On the other hand, three genes were up-regulated across all storage treatments (Fig. 4A). More pathogen receptor genes were down-regulated at 40 d compared to other storage times.

**Fig. 5:**
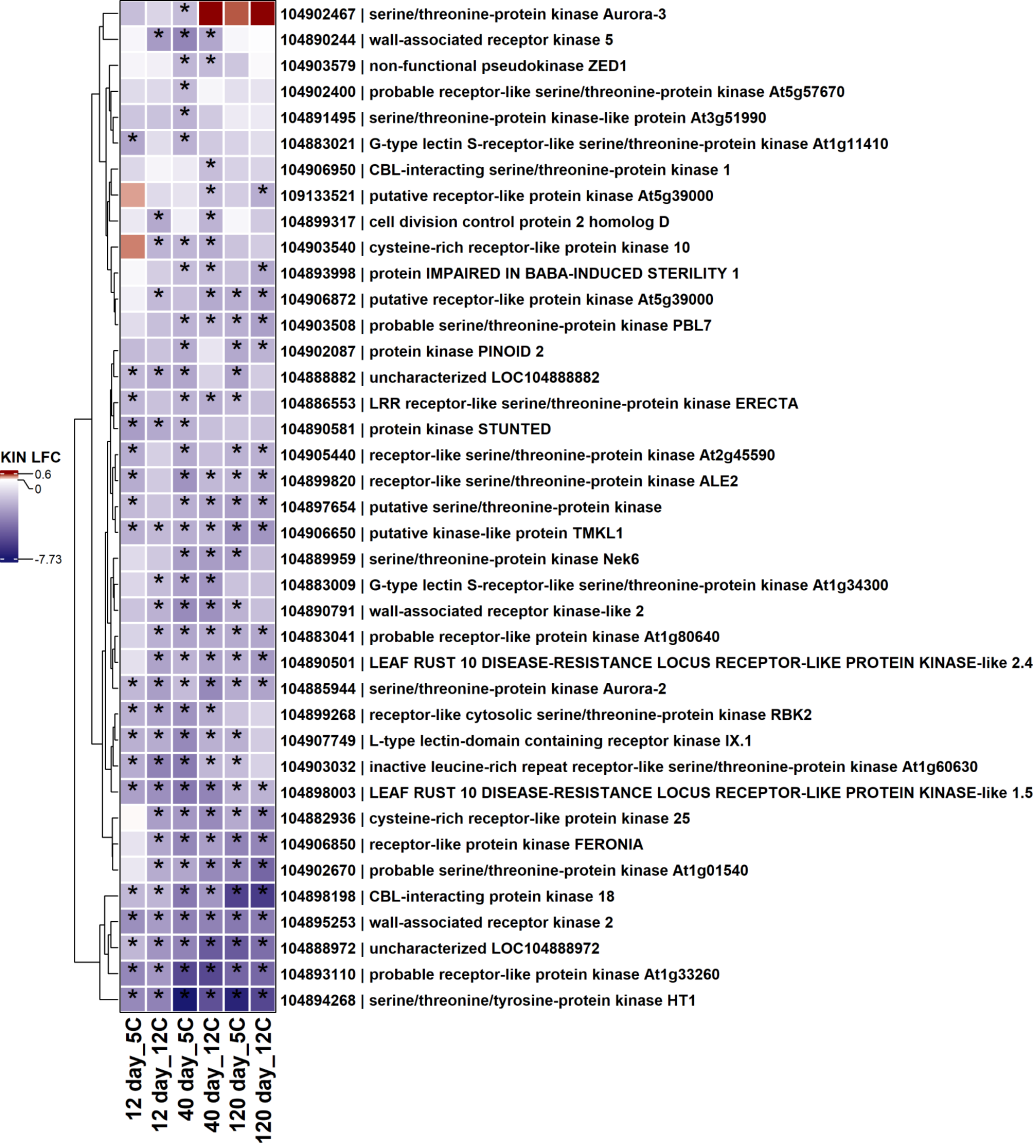
Heat map of down-regulated receptor genes with protein kinase domains (KIN) in postharvest sugar beet roots. The asterisk (*) indicates significantly expressed genes (absolute log_2_fold change > 2.0 and P-adj < 0.01). Squares are colored by differential expression status. LFC = Log_2_fold change.

**Fig. 6:**
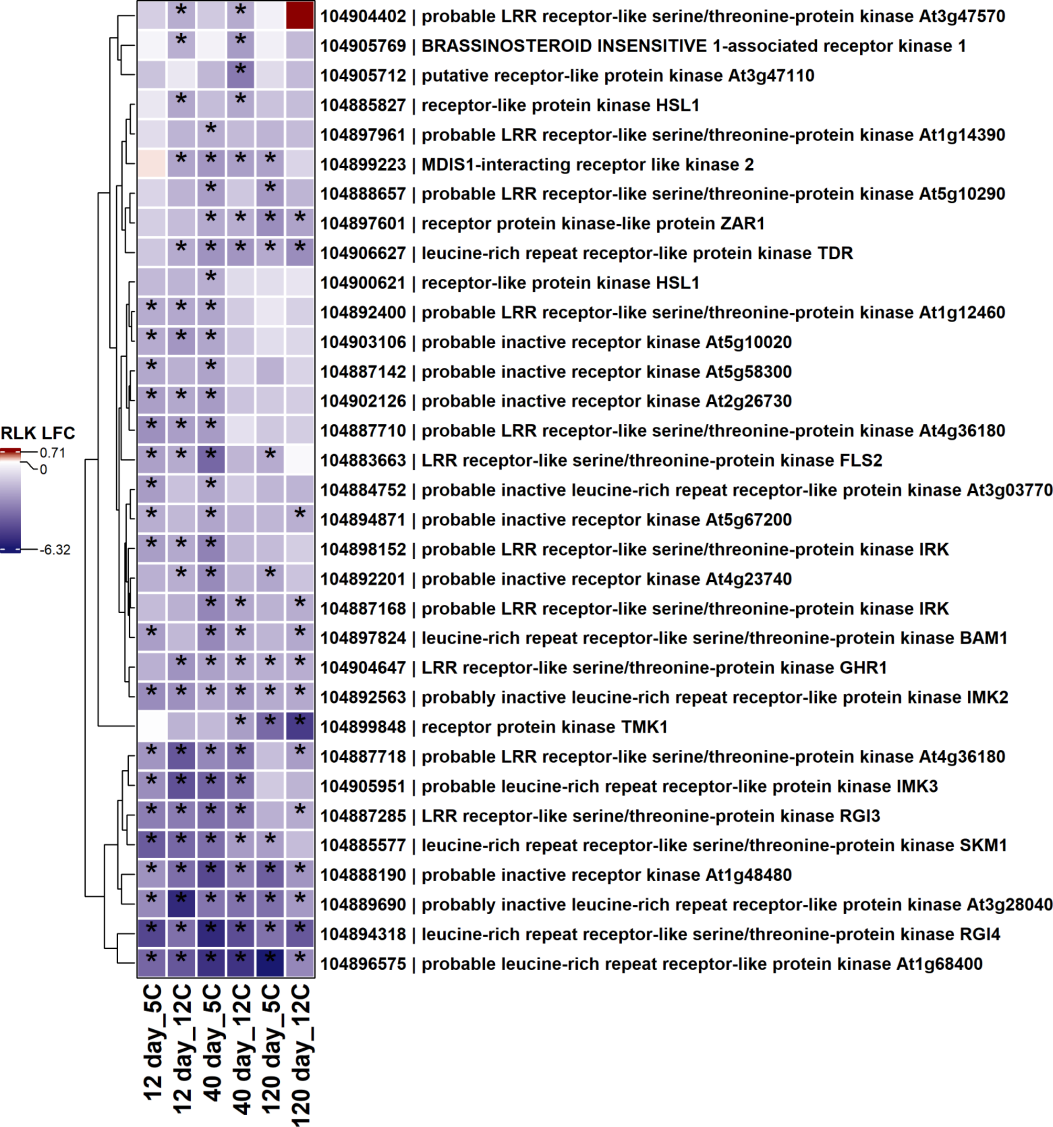
Heat map of down-regulated receptor genes with receptor-like protein kinase domains (RLK) in postharvest sugar beet roots. The asterisk (*) indicates significantly expressed genes (absolute log_2_fold change > 2.0 and P-adj < 0.01). Squares are colored by differential expression status. LFC = Log_2_fold change.

**Fig. 7:**
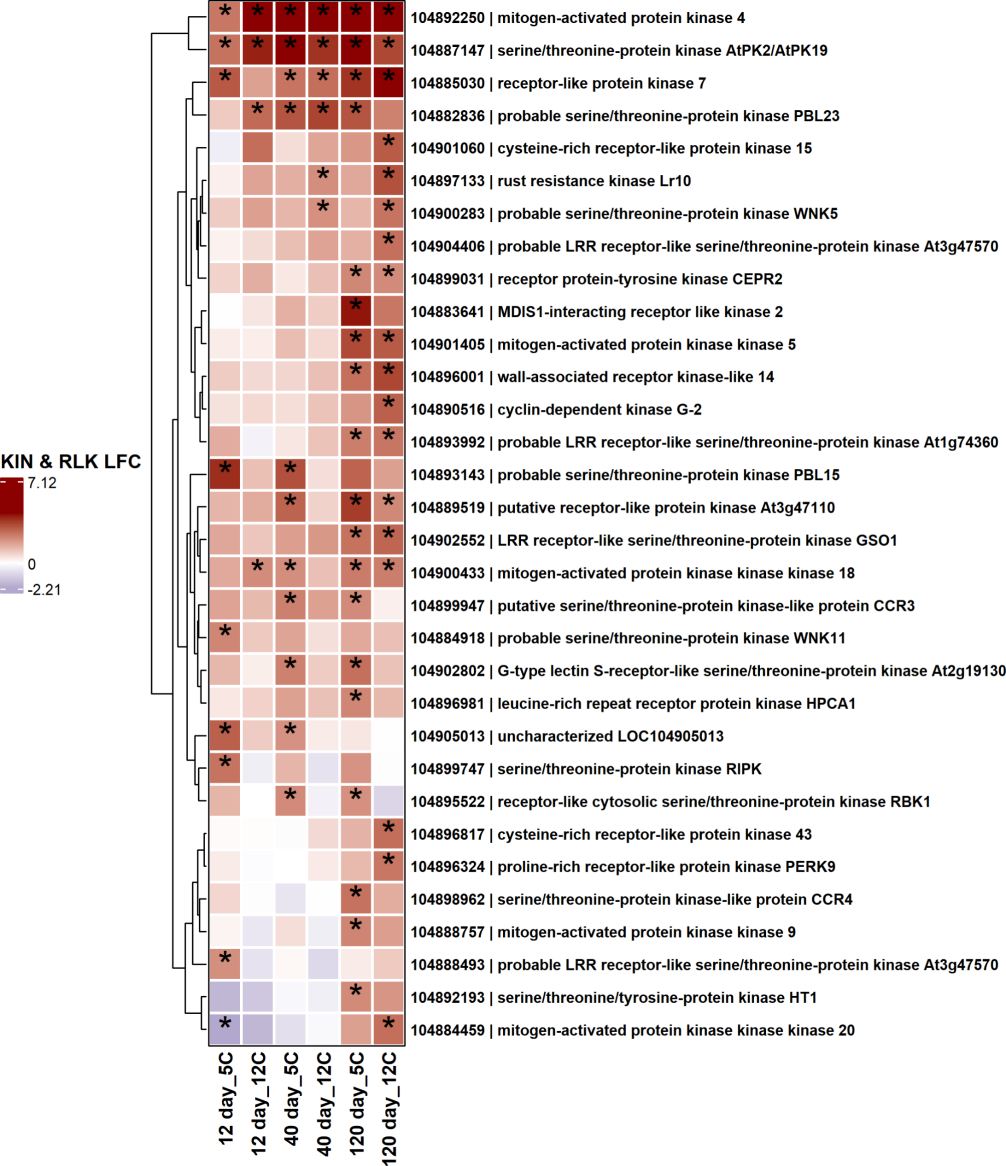
Heat map of upregulated receptor genes with protein kinase or receptor-like protein kinase domains in postharvest sugar beet roots. The asterisk (*) indicates significantly expressed genes (absolute log_2_fold change > 2.0 and P-adj < 0.01). Squares are colored by differential expression status. LFC = Log_2_fold change.

Among down regulated pathogen receptor genes, 39 genes with protein kinase domains and 33 genes with receptor-like protein kinase domains were down-regulated during storage (Figs. 5 and 6). Nine genes with a protein kinase or receptor-like protein kinase domain were down-regulated in all storage conditions. Genes related to leucine or probable leucine rich receptor or receptor-like kinases, wall-associated receptor kinases, and serine/threonine-protein kinases were major receptor genes significantly down-regulated during storage (Figs. 5 and 6). Fewer receptor genes containing protein kinase or receptor-like protein kinase domains were significantly up-regulated in response to storage (Fig. 7). Genes related to mitogen-activated protein kinase 4 (MAPK4) and serine/threonine-protein kinase AtPK2/AtPK19 were significantly up-regulated across storage treatments (Fig. 5). Other infrequent receptor genes with coiled-coil or lectin kinase domain were variably up or down-regulated during storage (Supplementary Fig. 1).

## Storage duration and temperature effects on expression of phytohormone signaling genes

**Fig. 8:**
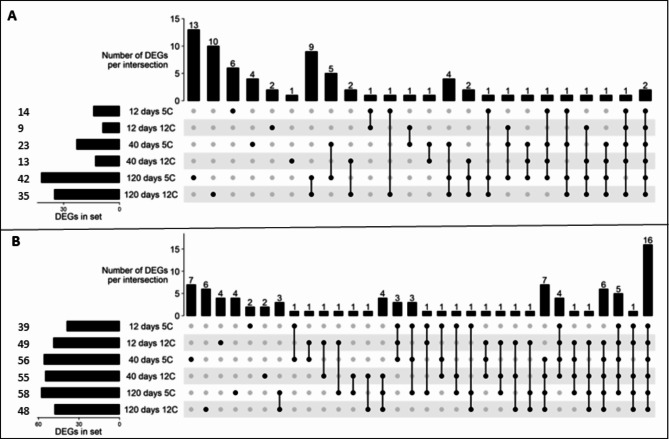
UpSet plots of significantly up (**A**) and down (**B**) regulated genes (absolute log_2_fold change > 2.0 and P-adj < 0.01) involved in phytohormone signal transduction pathways in stored sugar beet roots. Total counts of up-and down-regulated genes in each treatment were displayed as horizontal bars, located on the left side of each panel. Vertical black lines with filled dots indicate sets of intersections between two or more treatments. Vertical bars in the upper portion of each panel represent total counts of unique or shared genes across treatments. The list of up-and-down regulated genes was included in Supplementary File 2.

**Fig. 9:**
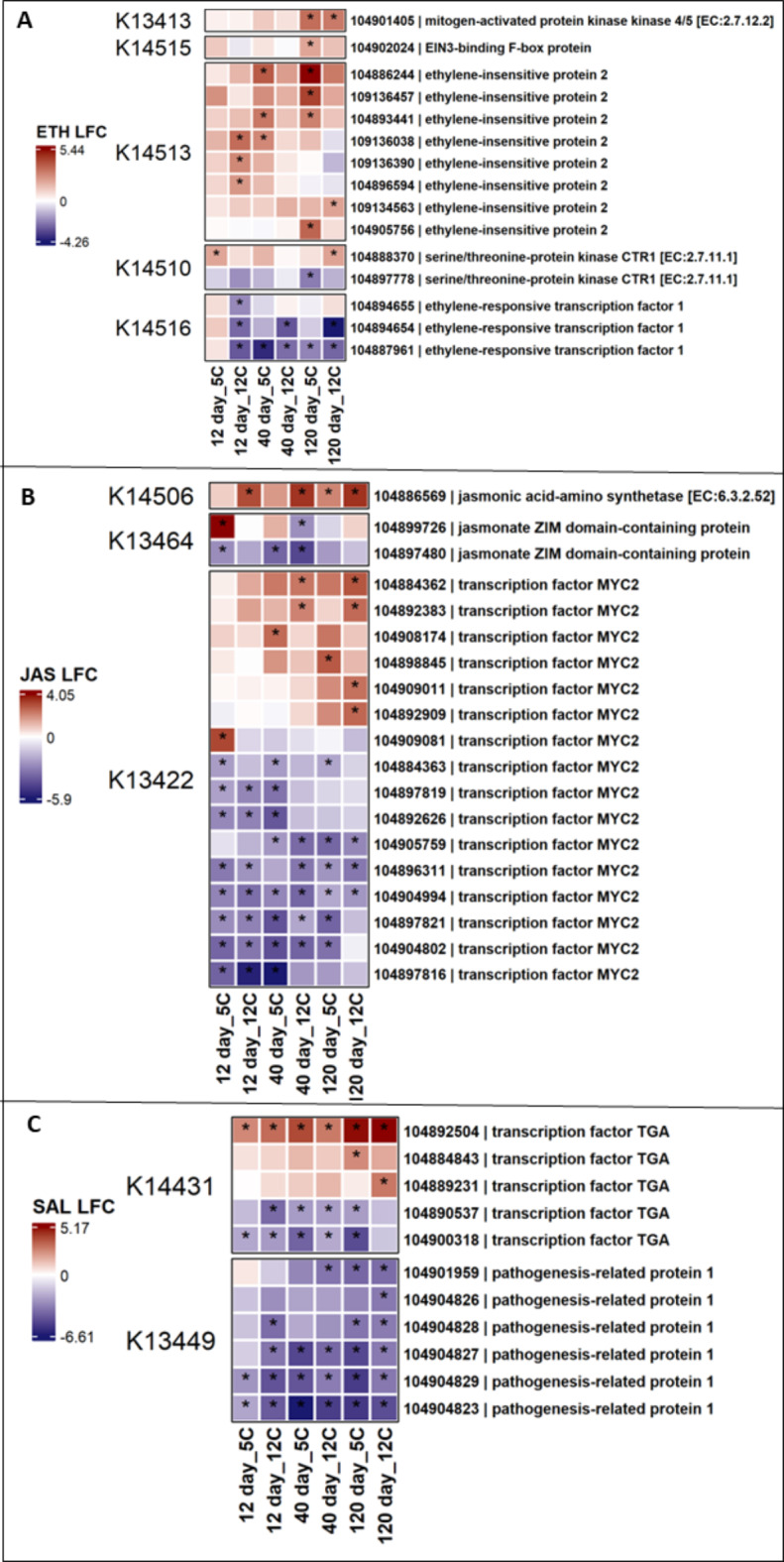
Heat map of differentially expressed genes involved in (**A**) ethylene, (**B**) jasmonic acid, and (**C**) salicylic acid hormonal signal transduction pathways in postharvest sugar beet roots. The asterisk (*) indicates significantly expressed genes (absolute log_2_fold change > 2.0 and P-adj < 0.01). Squares are colored by differential expression status. K13413….K3449 are KEGG orthology identifiers and indicate individual gene functions on the pathways. Genes having the same molecular function is annotated with the same KEGG identifier. For example, genes 104899726 and 104897480 were identified by KEGG as “Jasmonate ZIM-domain containing protein” and, for this reason, annotated with the same KEGG Identifier (aka KO) i.e. K13464.

A total of 275 differentially expressed genes involved in abscisic acid (ABA), auxin, brassinosteroid (BR), cytokinin (CTK), ethylene (ET), gibberellic acid (GA), jasmonic acid (JA), and salicylic acid (SA) signal transduction pathways were identified using the KEGG plant hormone database^42,43^. A total of 72 and 93 phytohormone signaling pathway genes were significantly up-and down-regulated (absolute log_2_fold change > 2.0 and P-adj < 0.01), respectively. The expression of phytohormone signaling genes was affected by both storage duration and temperature. However, storage time rather than storage temperature had a larger influence on the differential expression of signaling genes. In total, up-regulated genes increased their expression from 12 to 40 to 120 d of storage (Fig. 8A), while down-regulated genes increased from 12 to 40 d but remained similar at 40 and 120 d (Fig. 8B). Differentially expressed genes involved in ethylene, JA, and SA were examined further due to the well-defined role of these hormones in plant defense responses (Fig. 9)^22^. Multiple genes encoding ethylene-insensitive protein 2 (ethylene signaling pathway), transcription factor MYC2 (JA signaling pathway), and transcription factor TGA (TGACG MOTIF-BINDING FACTOR) (SA signaling pathway) were significantly altered in expression during storage. Some genes related to ethylene-responsive transcription factor 1 (ethylene signaling pathway), transcription factor MYC2 (JA signaling pathway), and pathogenesis-related protein 1 (PR-1) and transcription factor TGA (SA signaling pathway) were significantly down-regulated at one or more time points (Fig. 9). Several genes related to ABA, auxin, BR, CTK, and GA signaling pathways were also up-or down-regulated (Supplementary Figs. 2,3) in stored roots and are listed in the Supplementary File 2.

## Discussion

Storage diseases can cause significant sucrose loss and quality deterioration in postharvest sugar beet roots. *Botrytis cinerea* and *P. vulpinum* are major pathogens of stored sugar beet roots^7,8,14^. Understanding the biology of storage diseases and defense responses is vital to minimize sugar loss from postharvest sugar beet roots. In this study, effects of storage duration and storage temperature on disease susceptibility were assessed by inoculating roots with *B. cinerea* and *P. vulpinum* at different storage times. Additionally, changes in the expression of defense related genes were identified in sugar beet roots that may influence changes in innate immunity or susceptibility to these pathogens.

*Botrytis cinerea* is a necrotrophic pathogen which causes gray mold diseases and can infect more than 200 plant species, including sugar beet roots in storage^16,45^. In the process of suppressing host defenses and initiating the infection, this fungus secretes cell wall degrading enzymes and reactive oxygen species that degrade and kill host cells^16,17^. In our study, sugar beet roots inoculated with *B. cinerea* were found most susceptible at 40 d post storage compared to 0, 12 or 120 d (Fig. 2A) indicating that storage time can affect the susceptibility of roots to disease development and root deterioration. The underlying mechanism causing the higher tissue damage at 40 d is not known, although it is possible that the progressive cell wall changes occurring during storage could affect disease susceptibility and lesion spreading. Furthermore, at 40 d, significantly more root deterioration was observed at 12 °C as compared to 5 °C indicating that root susceptibility likely increases with an increase in storage temperature. Like *B. cinerea*, *P. vulpinum* is a polyphagous necrotrophic pathogen which can infect many postharvest crop commodities including sugar beet^7,14^. In sugar beet, *P. vulpinum* has commonly been isolated from infected roots in storage and is associated with root rotting and necrotic lesions with blue mold symptoms. In our disease assays, sugar beet roots stored for 40 and 120 d and subsequently inoculated with *P. vulpinum* showed significantly higher rot symptoms and tissue damage than 0 or 12 d of storage (Fig. 2B). The effect of temperature on the severity of rotting was only significant in roots stored for 120 d where higher rotted tissue was observed at 12 °C than 5 °C (Fig. 2B). Past studies were performed to understand physiological processes and enzymatic activities that lead to the sucrose losses in stored roots^5,46^, while this study provides new insights into how storage time and temperature affects the susceptibility or defensive capability of sugar beet roots against microbial pathogens. Our results suggest that storage time and storage temperature are critical determinants for increasing susceptibility of sugar beet roots to storage pathogens such as *B. cinerea* and *P. vulpinum.* Interestingly, rotting damage in *B. cinerea* inoculated roots was twice that of *P. vulpinum* inoculated roots, especially at 40 and 120 d, although no such discrepancy was observed in roots inoculated at 0 d storage (Fig. 2). Perhaps, *B. cinerea* is more aggressive and rapidly overcomes the defensive capability of roots that were stored for longer times, such as 40 and 120 d. Likewise, increased down-regulation of pathogen receptor genes at 40 and 120 d may imply declining of innate immunity in stored roots, which corroborates with disease progression with storage time.

Despite prior work on the genetics of host-pathogen interactions for foliar or root diseases in sugar beet^21,47^, little is known about defense-related transcriptional changes in sugar beet roots during storage. Pathogen receptor and phytohormone signaling genes have a significant role in plants to activate and confer defense responses against challenges from microbial pathogens^23–25^. In this study, transcriptional changes of pathogen receptors and phytohormone signaling genes during sugar beet storage were identified. Storage altered the expression of pathogen receptor genes as the genes expressed in freshly harvested roots (0 day) differed from stored roots under variable temperatures and times (Fig. 3). Storage caused hundreds of pathogen receptor genes to be differentially expressed, with distinct transcriptional responses to storage temperature and time found. Sugar beet roots undergo genetic and physiological changes during storage as observed in other crops which may lead to the transcriptional reprograming of expression of plant resistance genes^48,49^. Transcriptional reprograming began within a few days of storage as indicated by the large number of up- and down-regulated genes that were identified already at 12 d storage. As storage continued, a maximum in the number of pathogen receptor genes that were down-regulated occurred at 40 d which correlates with our findings of increased root susceptibility and maximum tissue damage. These results are consistent with the idea that immunity is compromised in stored sugar beet roots through the downregulation of resistance genes. Past studies also suggested that downregulation of immune receptor genes increases host susceptibility and disease damage^26,50,51^. Receptor genes that were down-regulated between 12 and 40 d are potential candidates for future research to understand the molecular basis of diminishing host resistance during storage as this is the time when susceptibility increases the most. On the other hand, some pathogen receptor genes were up-regulated in stored sugar beet roots which may be crucial to maintain defense activities and maintain root health during storage (Fig. 7). The upregulation of genes encoding for MPK4 and serine/threonine-protein kinase AtPK2/AtPK19 potentially activates signaling pathways and cytoskeleton organization in sugar beet roots as documented in other plants to preserve root health during storage^52,53^, but this awaits further validation.

Hormone signal transduction pathways are important in plant defense responses^23,54^ and many changes in genes of ABA, auxin, BR, CTK, ET, GA, JA, and SA signaling pathways were also noted. Overall, more phytohormone signaling genes were down-regulated versus up-regulated in stored sugar beet roots (Fig. 8). A similar pattern of down regulation of pathogen receptor and phytohormone signaling genes were identified, suggesting there was direct or indirect co-regulation of these genes. Ethylene, JA, and SA signal transduction genes such as transcription factor TGA, PR1 proteins, and transcription factor MYC2 were down-regulated which probably cooperated with down-regulated pathogen receptor genes to compromise the defensive capability of sugar beet roots during storage. Nevertheless, the upregulation of hormonal signaling genes may have a general effect on the ability of a root to defend itself during storage. Phytohormones such as ET, JA, and SA are considered as key modulators to induce resistance and improve storage life of many postharvest products^55^. The upregulation of ET, JA, and SA signal transduction genes can activate metabolic and signal transduction pathways leading to increasing cell wall rigidity and antioxidant enzyme activities, delaying senescence, and defensive gene expression during storage^56–58^. In sugar beet, postharvest jasmonate treatments stimulated accumulation of antimicrobial and antioxidant compounds, improved mechanical strengths of cell walls, and provided resistance to storage rots^33,59^. Future work on key regulators of mutual or exclusive interactions of pathogen receptor and phytohormone signaling genes during storage will be useful to improve the host resistance for managing storage diseases and minimize the sugar loss in sugar beet.

In this study, we demonstrated that the ability of roots to defend themselves against two major storage pathogens declines in storage. Both storage temperature and duration affected the decline in the ability of roots to defend themselves, although the effect of storage temperature was smaller than the effect of storage duration. Large and complex changes in plant defense and hormonal signaling genes occurred in response to storage. Taken together, this study provides an overview of defensive gene expression in sugar beet roots during storage, which can guide additional studies to assess candidate genes for resistance to storage pathogens and understand how the root’s innate defense system can be employed to improve storage. In future research, we will initiate studies to understand the functional significance of these genes to improve disease resistance and storage properties.

## Electronic supplementary material

Below is the link to the electronic supplementary material.

Supplementary Material 1

Supplementary Material 2

Supplementary Material 3

## Data Availability

The RNA-seq dataset generated in this study is available through the NCBI Sequence Read Archive under BioProject identification number PRJNA938134.
