# Supplementary material for: Sugar beet root susceptibility to storage rots and downregulation of plant defense genes increases with time in storage
Source: Sci Rep. 2024 Nov 8;14:27235. doi: 10.1038/s41598-024-78323-4 (PMC11549380; doi:10.1038/s41598-024-78323-4)
Supplement: Supplementary file 3 — Supplementary Material 3 [file 41598_2024_78323_MOESM3_ESM.docx]

**Supplementary Material**


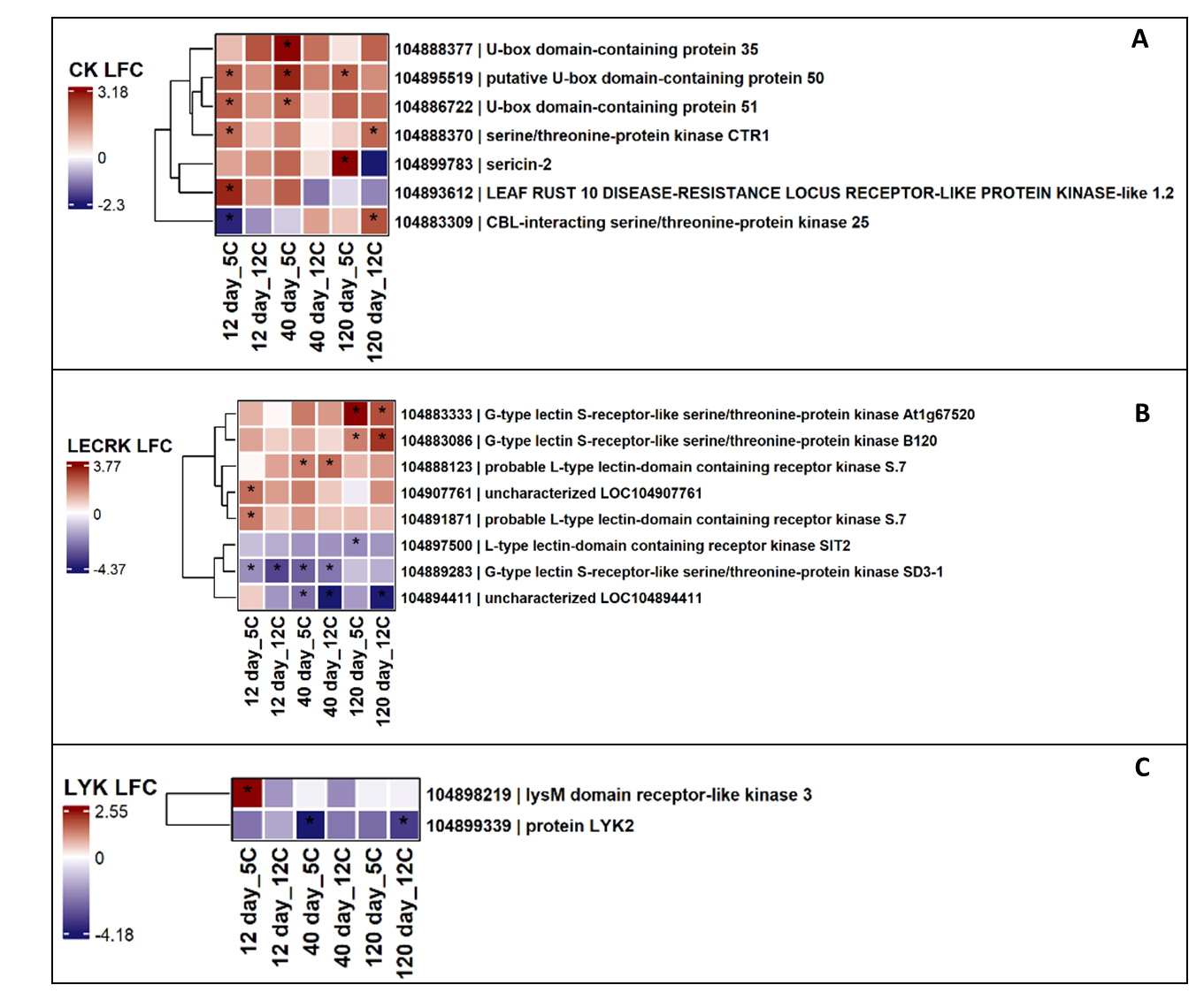


Supplementary Fig. 1. Heat map of differentially expressed pathogen receptor genes contain (A) coiled-coil and kinase domains (CK); (B) Lectin-like motifs and kinase domains (LECRK); and (C) lysin motif receptor-like kinase domains (LYK) involved in the host plant resistance process in sugar beet roots during storage under variable temperature and time. The asterisk (*) indicates significantly differentially expressed genes (absolute log_2_fold change > 2.0 and P-adj < 0.01). Squares are colored by differential expression status. LFC = Log_2_fold change.


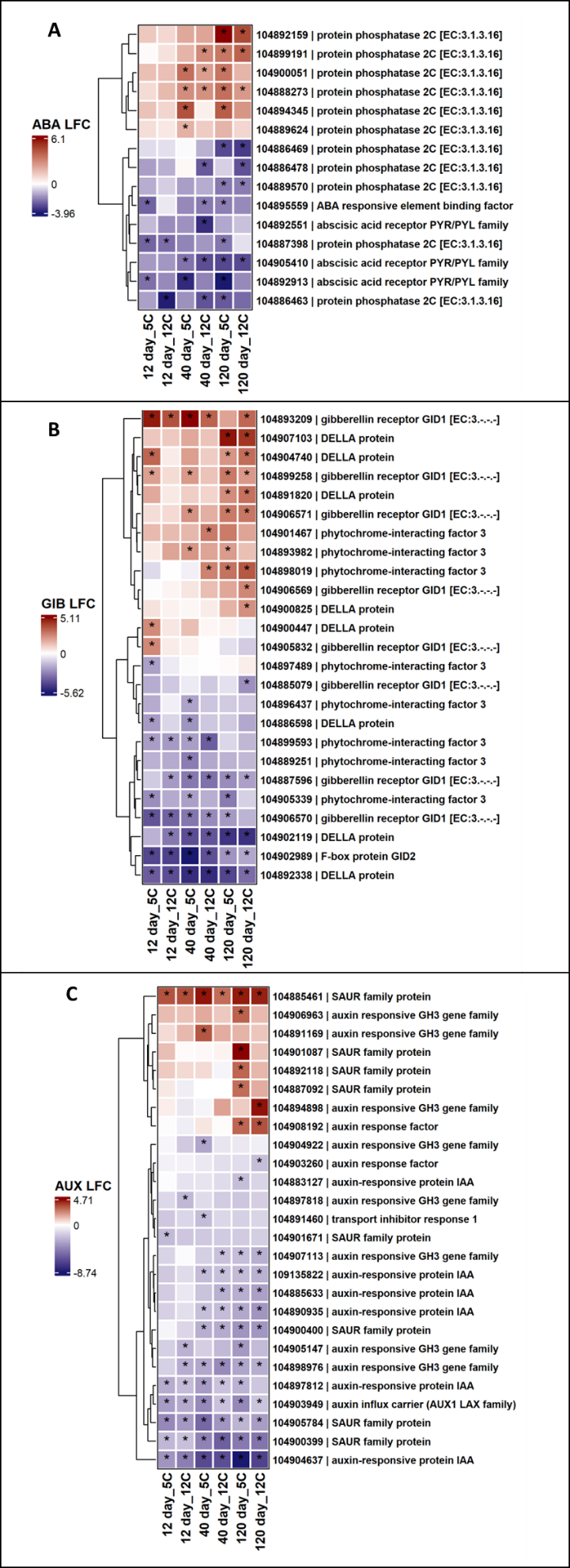


Supplementary Fig. 2. Heat map of genes involved in (A) abscisic acid, (B) gibberellic acid, and (C) auxin signal transduction pathways in sugar beet roots during storage under variable temperature and time. The asterisk (*) indicates significantly differentially expressed genes (absolute log2fold change > 2.0 and P-adj < 0.01). Squares are colored by differential expression status. LFC = Log_2_fold change.


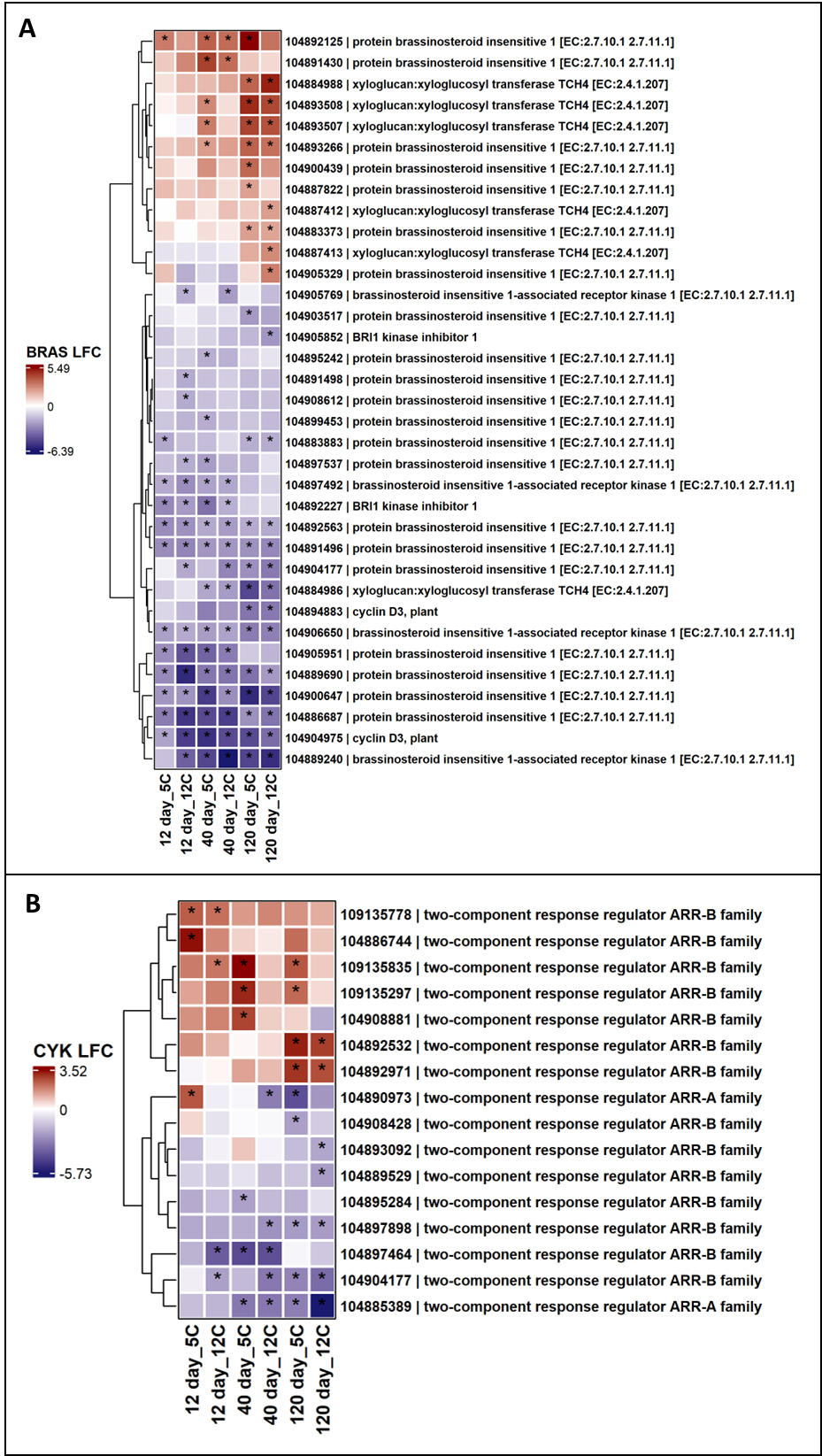


Supplementary Fig. 3. Heat map of genes involved in (A) brassinosteroid, and (B) cytokinin signal transduction pathways in sugar beet roots during storage under variable temperature and time. The asterisk (*) indicates significantly differentially expressed genes (absolute log_2_fold change > 2.0 and P-adj < 0.01). Squares are colored by differential expression status. LFC = Log_2_fold change.
